# Enhanced Monocular Visual Odometry Integrated with Laser Distance Meter for Astronaut Navigation

**DOI:** 10.3390/s140304981

**Published:** 2014-03-11

**Authors:** Kai Wu, Kaichang Di, Xun Sun, Wenhui Wan, Zhaoqin Liu

**Affiliations:** 1 State Key Laboratory of Remote Sensing Science, Institute of Remote Sensing and Digital Earth, Chinese Academy of Sciences, Beijing 100000, China; E-Mails: wukai@radi.ac.cn (K.W.); wanwh@radi.ac.cn (W.W.); liuzq@radi.ac.cn (Z.L.); 2 Computer and Information Engineering College, Henan University, Kaifeng 475000, China; E-Mail: sunxun2012@163.com

**Keywords:** monocular visual odometry, laser distance meter, scale drift, calibration, navigation

## Abstract

Visual odometry provides astronauts with accurate knowledge of their position and orientation. Wearable astronaut navigation systems should be simple and compact. Therefore, monocular vision methods are preferred over stereo vision systems, commonly used in mobile robots. However, the projective nature of monocular visual odometry causes a scale ambiguity problem. In this paper, we focus on the integration of a monocular camera with a laser distance meter to solve this problem. The most remarkable advantage of the system is its ability to recover a global trajectory for monocular image sequences by incorporating direct distance measurements. First, we propose a robust and easy-to-use extrinsic calibration method between camera and laser distance meter. Second, we present a navigation scheme that fuses distance measurements with monocular sequences to correct the scale drift. In particular, we explain in detail how to match the projection of the invisible laser pointer on other frames. Our proposed integration architecture is examined using a live dataset collected in a simulated lunar surface environment. The experimental results demonstrate the feasibility and effectiveness of the proposed method.

## 1. Introduction

The astronaut navigation system is one of the most important systems for manned missions on the lunar surface, as it keeps astronauts safe while exploring previously unknown environments and provides accurate positions for scientific targets. Although the principle of the lunar astronaut navigation system is much the same as that of a pedestrian navigation system, the global positioning system (GPS)-denied environment, and the absence of a dipolar magnetic field and an atmosphere limits the application of several traditional sensors that have been successfully used for pedestrian navigation on Earth, such as GPS, magnetometers and barometers [1]. Furthermore, unlike lunar or Mars exploration rovers, the size, weight, and power of on-suit astronaut navigation sensors are strictly limited. Therefore, vision sensors are well suited for this type of navigation system, as they are light and power-saving. They can work effectively as long as there are enough textures that can be extracted.

Visual odometry (VO) is the process of incrementally estimating the pose of an agent from the apparent motion induced on the images of its onboard cameras. Early research into VO was devoted to solving the wheel slippage problem in uneven and rough terrains for planetary rovers; its implementation was finally successfully applied onboard the Mars rovers [2–4]. It is fascinating to see that it provides the rover with more accurate positioning compared to wheel odometry. Later Nister [5] proposed the first long-run VO implementation with a robust outlier rejection scheme. This capability makes it vitally important, especially in GPS-denied environments such as the lunar surface. However, most of the research in VO has been performed using a stereo vision scheme, which is certainly not an optimal vision configuration for an ideal wearable astronaut navigation system, because it is less compact and less power-saving compared to monocular vision. In this case, the stereo vision scheme becomes ineffective and should be substituted by monocular VO. More compact navigation systems [6] and successful results have been demonstrated using both omnidirectional and perspective cameras [7,8]. Closely related to VO is the parallel research undertaken on visual simultaneous localization and mapping (V-SLAM). This aims to estimate both the motion of an agent and the surrounding map. Most V-SLAM work has been limited to small or indoor workspaces [9,10] and also involved stereo cameras. This approach is generally not appropriate for large-scale displacements because of algorithmic complexity and growing complexity [11]. Recently, great developments have been made by Strasdat [12] using only monocular image input after adopting the key-frame and Bundle Adjustment (BA) [13] optimization approaches of the state-of-the-art VO systems.

Due to the nature of monocular systems, with bearing information only available in a single frame, geometry must be inferred over time and 3D landmarks cannot be fully constrained before observations from multiple viewpoints can be made. Furthermore, there is the difficulty that the absolute scale cannot be obtained in a single frame and motion can only be recovered up to a scale factor. This absolute scale cannot be determined unless absolute scale information about the real world is introduced into the system. Without extra measurements, the scale is less constrained and error accumulates over time while motion is integrated from frame-to-frame estimation. This is the scale ambiguity problem for monocular VO. Special attention has been paid to this issue recently and a number of solutions have been proposed to solve the undetermined scale factor. Scaramuzza [14] used the height of the camera from the ground plane to obtain the global scale factor. Additionally, an observation of the average speed of the vehicle is also proposed to constrain the displacement of the camera [15]. While these techniques may become popularly used in monocular VO for vehicles, the motion constraints of a steady state may not work out for astronaut navigation. Also, by including additional carefully measured objects in the scene during the initialization stage, such as a calibration object, a metric scale can be fixed [9]. However, this metric scale is liable to drift over time. The pose-graph optimization technique presented by Strasdat [12] resolves the scale drift only at loop closures. A commonly used approach called sliding window bundle adjustment has been demonstrated to decrease the scale drift [16]. In some other work, extra metric sensors, such as inertial measurement units (IMU) and range sensors were also introduced to compensate for scale drift [17,18].

The integration of a camera and a laser distance meter (LDM) was first proposed by Ordonez [19] and was applied for 2D measurement of façade window apertures. In that work, Ordonez presented in detail the extrinsic calibration method of a digital camera and a LDM. Later, this low-cost 2D measurement system was extended to reconstruct scaled 3D models of buildings [20].

The issues mentioned above motivated us to use a monocular camera as the main sensor, aided by LDM for scaled navigation. However, as was admitted by the author in [20], there is a limitation that the shots must obey a plane constraint and the laser spot of the distance meter must fall in contact with a planar surface. Meanwhile, the process of the extrinsic calibration method of the camera and the laser distance meter proposed above is not simple and robust, as it requires careful intervention from the user, such as manual selection of the laser pointer's projection center.

In this paper, we focus on the integration of a laser distance meter and a monocular camera for applications such as astronaut navigation. We solve the scale ambiguity problem using metric measurements from a laser distance meter. Nevertheless, unlike 2D laser range finders and 3D laser scanners, which are widely used in the robotics community and provide both range and angular information on a series of laser points, LDM provides only the distance of a single laser dot. Therefore, compared with 2D laser range finders or 3D laser scanners, LDM consumes less power and simplifies the algorithm pipeline when integrated with a camera, as only one pixel of the image contains depth information. Besides, LDM has a more distant range to work in. So far most research concerning integration of a LDM and a camera has been for 3D model measurement or reconstruction, but not for scalable monocular VO. The main contribution of this work is the proposal of a novel enhanced monocular VO scheme by imposing an absolute scale constraint through integrating measurements from the LDM.

First, to obtain more accurate metric scaled navigation results, a flexible and robust extrinsic calibration method between the camera and the LDM is presented. This whole calibration process requires almost no manual intervention and is robust to gross errors. As soon as extrinsic calibration of the system is completed and geometrical parameters are ready, a global scaled monocular VO pipeline is proposed. We particularly describe how to match the invisible laser spot on other frames in detail and how to correct the scale drift using distance measurement and calibration parameters.

In principle, this enhanced monocular VO method is certainly applicable for a mobile robot (e.g., a rover). However, stereo vision systems are commonly used in mobile robots due to its less limitation of the size of the navigation payload. In addition to navigation, stereo vision also offers stereo images, which are very valuable for understanding of the surrounding environment and investigation of the interested targets. Thus, in general stereo VO is more favorable than monocular VO for mobile robots.

This paper is organized as follows: Section 2 gives a general description of the system's device components and the working principle of our global scaled monocular VO scheme. Section 3 and Section 4 present extrinsic calibration and robust motion estimation with LDM and a monocular camera. Section 5 gives expanded results with real outdoor data in a simulated lunar surface environment. Finally, conclusions are given in Section 6.

## 2. Proposed Approach for Monocular Visual Odometry

**Figure 1.**
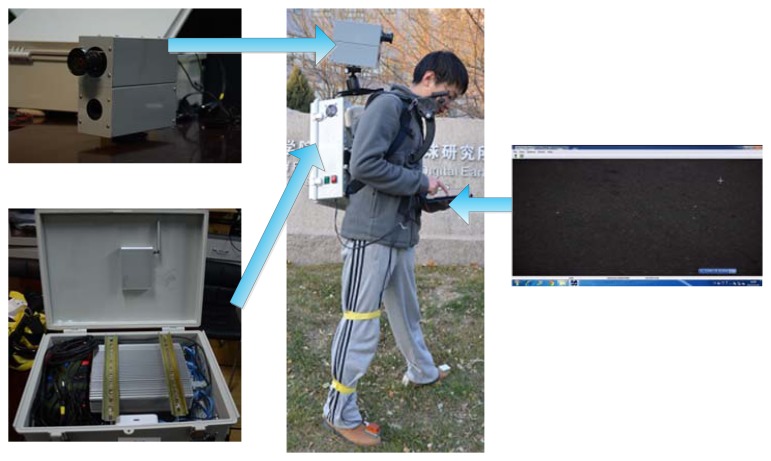
The hardware scheme for the astronaut navigation system.

The hardware of the astronaut navigation system consists of five components: an industrial camera (MV-VE141SC/SM, Microvision, Xi'an, China; image dimension: 1392 pixels × 1040 pixels, focal length: 12 mm, max. frequency: 10 Hz), a LDM (CLD-A with RS232 port, Chenglide, Beijing, China; accuracy: ±2 mm, max. frequency: 4 Hz), a specially designed platform for holding these two devices rigidly and provision of power from the on-suit batteries, an industrial computer (CPU: Intel core i5) to control the acquisition of images and laser readings, and an iPad to control the computer triggering the signal to the camera and the distance meter through a local Wi-Fi network. Figure 1 shows the hardware components of our navigation system and the right-hand part of the figure shows the screen of the iPad while taking images.

**Figure 2.**
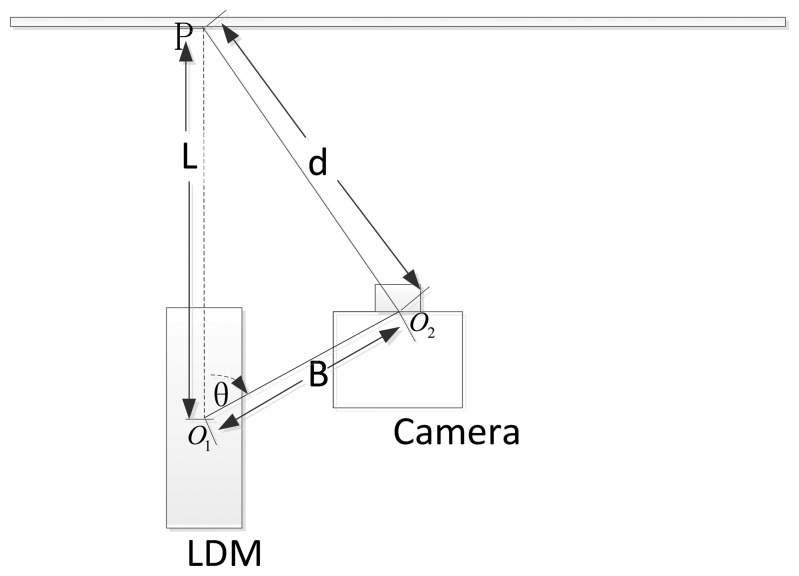
Geometrical relationship of the LDM and the camera.

The laser beam is collinear, so it can be modeled by a straight line. In Figure 2, a view of the navigation system with its mathematical model is shown for clarity. The system calculates the distance *d* between the optical center *O*_2_ and the laser pointer *P* by knowing the distance *L* from *P* to the LDM's origin *O*_1_ measured with the LDM, and the geometrical relationship between the camera and the LDM. As we are only interested in the distance *d*, in this paper, the geometrical relationship between the distance meter and the camera is modeled by just two parameters:

- The distance *B* from the LDM's origin to the optical center of the camera
- The direction angle *θ* between the laser beam and the direction from *O*_1_ to *O*_2_.

As illustrated in Figure 2, the following expression can be deduced based on the triangulation principle:

(1) $$d=\sqrt{B^{2}+L^{2}-2\cdot B\cdot L\cdot \cos\theta }$$

These two parameters, *B* and *θ*, are known after extrinsic calibration that will be described in detail in the following section.

Meanwhile, for each laser pointer reading taken, the point has its projection on its synchronized image which is difficult to detect, as it is mostly invisible on daytime images because the contrast with the environment is too low. Its position can be determined by searching in an index table, which can be created at night by taking a series of images with varying distances and detecting the laser pointer projection center. This index table describes the one-to-one relationship between the distance measured by the LDM and the image position projected on the image. In other words, once we know the distance of the laser pointer measured by the LDM, we can calculate its distance to the camera center and its projection position on the synchronized image taken at the same time. This extra global scale information can be incorporated into monocular VO to restrict scale drift effectively.

**Figure 3.**
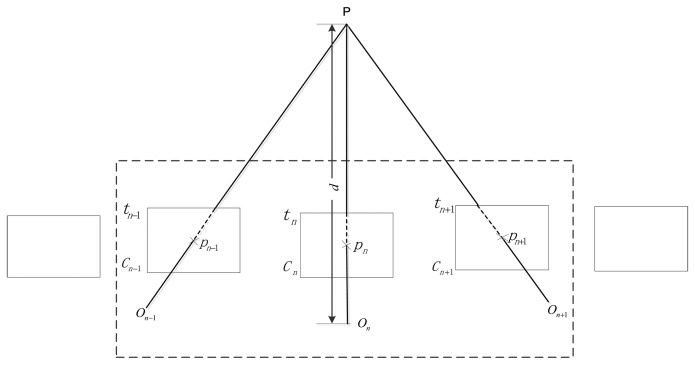
The enhanced scale drift free monocular VO system.

Figure 3 illustrates the nature of the scale ambiguity of the monocular system and the principle of our enhanced monocular system. The camera position can slide an unknown amount along the translation direction and estimation of the camera pose is intrinsically scale free. However, if we can track the laser pointer *P* on frame *C*_n−1_ and *C*_n+1_ successfully, we can obtain its depth at time *t*_n_ in this stereo model by triangulation. As we can calculate its global scale depth d with Equation (1) at time *t*_n_ using the distance measured, we can scale this stereo model with no drift. In this way, the scale drift is corrected whenever tracking along nearby key-frames is successful.

**Figure 4.**
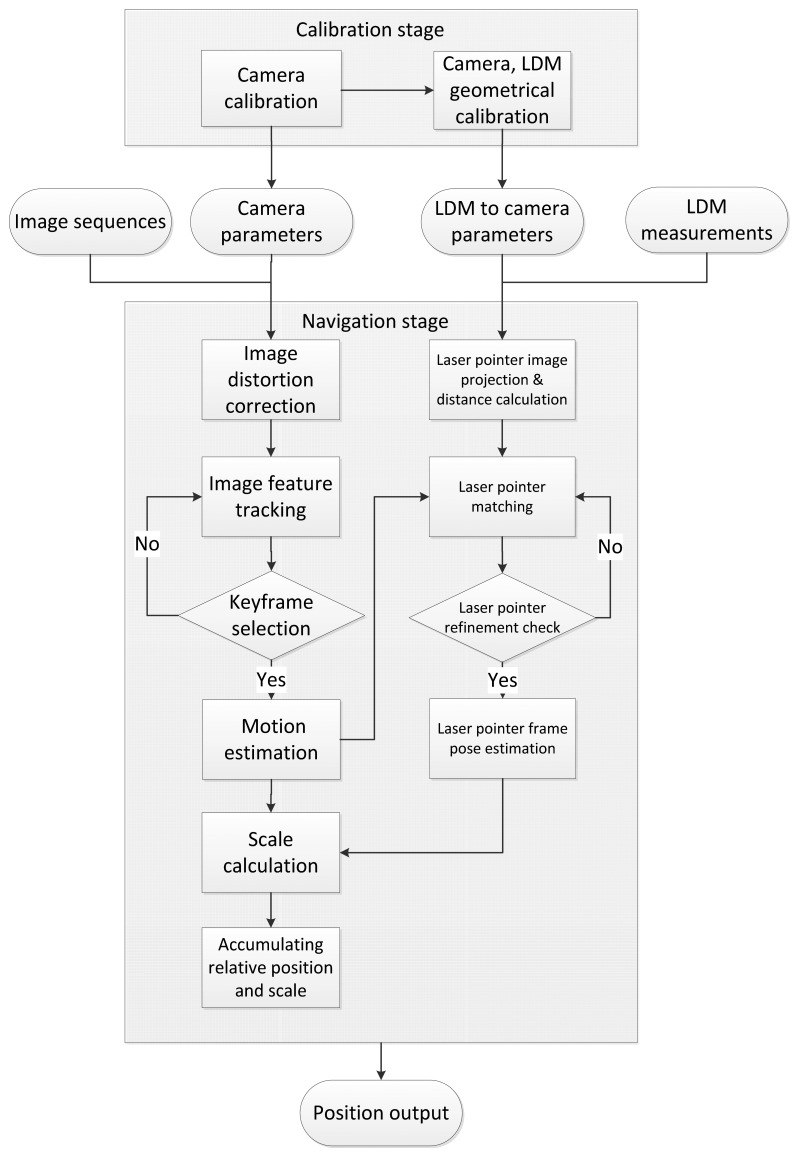
Flowchart of the enhanced monocular VO system pipeline.

In this paper, this astronaut navigation system can be divided into two parts: the calibration stage and the navigation stage, as illustrated in Figure 4. As quality of calibration is crucial to ensure accurate estimation of motion displacement, we propose a robust method to implement extrinsic calibration of the camera and the LDM. In particular, we create an index table to establish directly the relationship between the distance measurements from the LDM and its projection position on the synchronized image, further simplifying the calibration process and reducing the systematic error.

After finishing this preparation stage, the navigation stage is begun using our enhanced VO method. As the quality of image tracking is important for obtaining a robust and accurate estimate [5,15], we use the principle that key-points should cover the image as evenly as possible. Image features are tracked along image sequences and only a subset of them, called key-frames, are selected for motion estimation. In previous work, a key-frame selection scheme was proposed and only frames in good state for triangulation were selected [11]. Our proposal follows this scheme and key-frames are selected for a further core computation step, named motion estimation, and the relative geometrical relationship of the image pair can be constructed. In the meantime, the laser pointer projection is matched on nearby key-frames for triangulation to obtain its relative distance to the camera. In this way, scale drift is corrected when this laser pointer is constrained by the global scale distance calculated from Equation (1).

Most laser pointers are projected on some image position with a weak feature response, thus it is difficult to find its correspondence directly on other images, a coarse-to-fine matching technique is proposed using local disparity constraints followed by dense matching, reducing the possibility of false matches in these local feature-less regions. When the laser pointer is matched successfully, the global scale can be recovered, as illustrated in Figure 3. Otherwise, a relative scale is calculated by exploiting the distance constraints between two image pairs [21]. By incorporating global scale constraints on monocular VO, we can effectively reduce the scale drift which accumulates quickly over a certain number of frames.

## 3. Robust Calibration of the Navigation Platform

The system must be calibrated before it can be used for measurement. Calibration of the system includes camera calibration and extrinsic calibration of the camera and the LDM. First we need to calibrate the camera; we use the commonly used Bouguet method [22]. A flat panel with grid corners as control points is required. For extrinsic calibration of the camera and the LDM, Ordonez [19] proposed a calibration method for this combined camera and LDM setup, and the relative orientation of the LDM to the camera is represented as a position vector and an angular unit vector. However, the experiment involves manual selection of the laser pointer projection center and uses only two laser pointer projections, which is not particularly accurate or robust. To increase the accuracy and robustness, we propose a two-step extrinsic calibration method using the principle of the RANSAC scheme. In the following sections, the detailed extrinsic calibration procedure will be introduced.

### 3.1. Detection of the Laser Pointer Projection Center

As noted by Ordonez [19], the precise position of the laser center is defined to be the point that is closest to the starting point. When projected to the synchronized image, this is the center of the brightest region. In our experimental setup, this device is placed in a dark environment, as this facilitates isolation of the laser pointer projection from surrounding environment. The system is initially set facing toward a wall with the measuring axis of the laser meter parallel to the normal ray of the plane surface. The procedure used involves taking a sequence of pictures with synchronized distance measurements while the system is slowly moved toward the wall.

**Figure 5.**
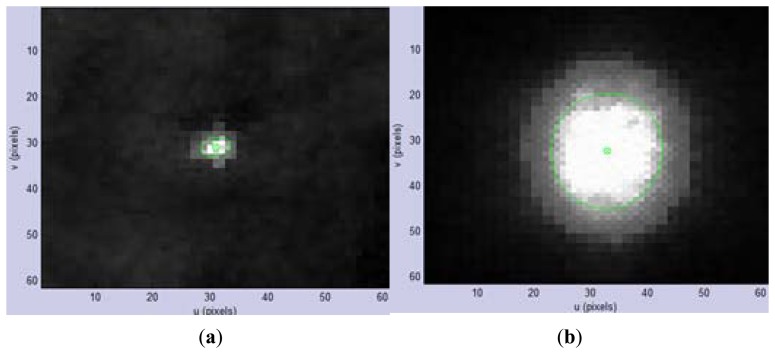
(**a**) Enlarged picture around the detected center at a distance of 5 m from the wall; (**b**) Enlarged picture around the detected center at a distance of 0.5 m from the wall.

As we can see from Figure 5a,b, the brightest region changes in size with varying distance, and its boundary is not regular. To detect the laser pointer center effectively, binary classification is first applied to the image, see Equation (2):

(2) $$c[u,v]=\{\begin{array}{ll} 0 & I[u,v]\leq t\\ 1 & I[u,v]>t \end{array},\forall (u,v)\in I$$

where *t* is the threshold for this binary classification, calculated by maximizing the variances between the classes of pixels below and above the threshold in reference to Otsu's threshold selection method [23], which works well for images with a bimodal intensity histogram. It's worth noting that quantization noise can be introduced during binarization and may affect the laser pointer center calculation. However, by taking this optimal threshold selection, the effects of binarization can be minimized for detection of the laser pointer center, by making the quantization noise satisfying normal distribution.

After this binarization, a sequence of operations involving erosion then dilation, known as opening, is performed to isolate a particular component before boundary detection, see Equation (3):

(3) I∘S=(IΘS)⊕S

Here we define the structuring element *S* as a 3 × 3 square region, Θ denotes the erosion operation on image *I* while ⊕ denotes the dilation operation on *I*. A concise description of the size, position, and shape of this component is further performed by using moments, which are a rich class of image features describing region size and location. The moments of an image are a scalar defined as Equation (4), where (*p* + *q*) is the order of the moment:

(4) $$m_{pq}=\sum_{(u,v)\in I}u^{p}v^{q}I[u,v]$$

Here the moments [24] are given as a physical interpretation by regarding the image function as a mass distribution and the laser pointer projection center is regarded as the center of the total mass of the region calculated as Equation (5):

(5) $$u_{c}=\frac{m_{10}}{m_{00}},v_{c}=\frac{m_{01}}{m_{00}}$$

**Figure 6.**
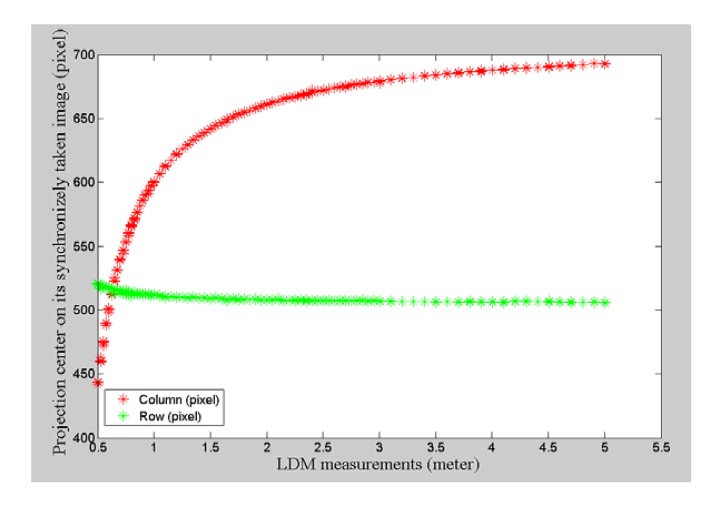
The image position of the projection center with varying laser distance measurements.

Figures 5a,b show the final detection results, with the inner circle overlying the laser pointer projection center. The projection center is precisely located within the illuminated region whether the area is large or small. Figure 6 illustrates the relationship between the projected image position of the laser pointer with distance measurements taken synchronously. To increase robustness for fixed distance measurement, multiple observations are made.

It can be inferred from Figure 6 that the laser pointer projection moves with a slower velocity on the image when the distance measured increases gradually, especially when the working distance is greater than 2 m. This characteristic is important because we can directly obtain the image position of the laser pointer from its distance measured through interpolation from a few reference points prepared in a nighttime environment. Fortunately, the working range is generally more than 2 m in our experiment.

### 3.2. Extrinsic Calibration of the Laser Distance Meter and the Camera

The above automatic laser pointer projection center detection algorithm produces a correspondence list *S* = {*L_i_* ∣(*x_i_*,*y_i_*, *i* = 1,2,3, …*N*} by associating the distances of many laser points with corresponding synchronized image projection centers. An index table can be created by selecting inliers from *S*. Therefore, for any distance measurement, we can obtain the projection center of the laser pointer on its synchronized image. With this step, we can calculate the extrinsic parameters by taking several shots in front of a grid panel.

#### 3.2.1. Laser Pointer Projection Based on the Index Table

Theoretically, all the laser pointer projections, although having varying distances, should lie on the same line on the image when the distance meter is rigidly attached to the camera. An exception is that the laser pointers are all projected onto the center of the projection plane regardless of its distance from the camera when the laser beam coincides with the lens axis of an ideal camera. However, this exception can be avoided for a real device configuration. Generally, both camera distortions and errors in the laser pointer center detection contribute to the final offsets of the line. By using the camera distortion coefficients [k_1_,k_2_,k_3_,p_1_,p_2_], the laser pointer image coordinates are all corrected to remove camera distortion effects. Formula (6) describes these distortion effects:

(6) $$\{\begin{array}{c} u^{d}=u+\delta_{u},v^{d}=v+\delta_{v},r^{2}=u^{2}+v^{2}\\ \left(\begin{array}{c} \delta_{u}\\ \delta_{v} \end{array}\right)=\left(\begin{array}{c} u(k_{1}r^{2}+k_{2}r^{4}+k_{3}r^{6})\\ v(k_{1}r^{2}+k_{2}r^{4}+k_{3}r^{6}) \end{array}\right)+\left(\begin{array}{c} 2p_{1}uv+p_{2}(r^{2}+2u^{2})\\ p_{1}(r^{2}+2v^{2})+2p_{1}uv \end{array}\right) \end{array}$$

where (*u^d^,v^d^*) is the original image coordinate, (*u,v*) is the image coordinate after distortion offsets are corrected and *r* is the radial distance from the principal point to the image point.

**Figure 7.**
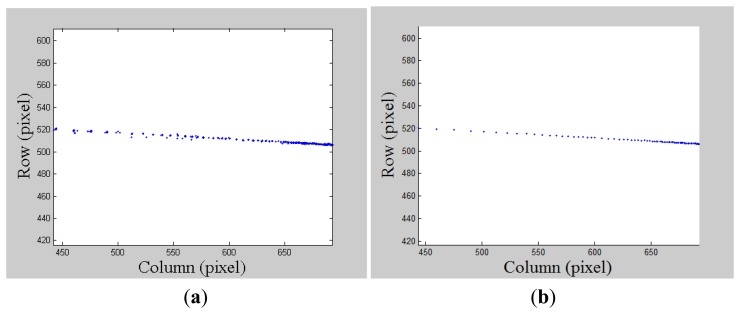
(**a**) Laser pointer projections on an image; (**b**) Laser pointer projections on an image with gross error removed after MLESAC.

The next step is to model this line by using undistorted laser pointer image coordinates projected from a sequence of measurements ranging from far to near. Problems such as this can be transformed into issues of parameter estimation. It can be clearly seen from Figure 7a that these laser pointer image projections are also contaminated by gross errors. We now focus on a line fitting model to introduce the robust estimate techniques.

Assuming the blue data set is {(*x_i_*,*y_i_* ∣ *i* = 1,2,3,…*N*}, for each point we wish to minimize the absolute value of the signed error:

(7) $$e_{M}(d;\theta )=\frac{ax+by+c}{\sqrt{a^{2}+b^{2}}}$$

Here, the parameter vector *θ* ∈ *R*^2^ describes the line *ax* + *by* + *c* = 0 (this is the model that we use to fit the measurements). We also model the fitting error as a Gaussian random variable with zero mean and standard deviation *σ_n_*, *i.e.*, *e_M_* (*d*;*θ*):N(0,*σ_n_*). A maximum likelihood approach implemented by MLESAC [25] (RANSAC's variant) is taken to find the parameter vector [*a*,*b*,*c*] that maximizes the likelihood of the joint error distribution:

(8) $$L(\theta )=p[e_{M}(d_{1};\theta ),\ldots ,e_{M}(d_{N};\theta )]$$

In our implementation, this standard deviation *σ_n_* is set to be 0.3 pixels. Figure 7b illustrates the status of the laser pointer projection with outliers removed by running MLESAC. While we obtain an initial estimate of the line's parameter vector, these parameters can be further refined by running nonlinear optimization using all the inliers. In our experiment, the squared mean error is 0.02 pixels for all the inliers after running optimization; thus, an accurate line model is recovered.

**Figure 8.**
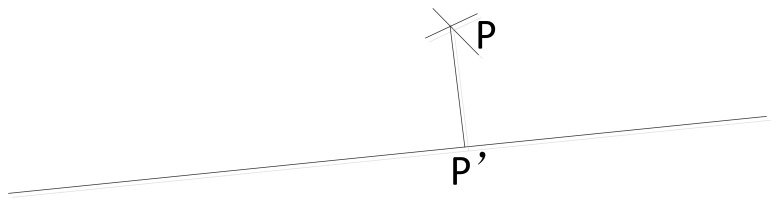
Laser pointer projection on the estimated line.

In Figure 8, supposing that *P* is an inlier, then {*L_i_* ∣(*x_i_*,*y_i_*, *i* = 1,2,3,…*N*} projected from *P* on the refined line is finally chosen as the laser pointer position on the image.

An index table *S* = {(*L_i_*,*x_i_*,*y_i_*)∣*i* = 1,2,3,…*N*} is created that directly establishes the relationship between the distance measurements and the laser pointer position on images by using all the inliers. In this way, for any measurement *L* returned by the LDM, we can find its nearby reference points *P_i_*(*L_i_,x_i_,y_i_*), *P_i_*_+1_(*L_i_*_+1_*,x_i_*_+1_*,y_i_*_+1_) from the index table and obtain its associated projection *P*(*x*,*y*) by interpolating from *P_i_*, *P_i_*_+1_, thus:

(9) $$\{\begin{array}{c} x=((L-L_{i})•x_{i+1}+(L_{i+1}-L)•x_{i})/(L_{i+1}-L_{i})\\ y=((L-L_{i})•y_{i+1}+(L_{i+1}-L)•y_{i})/(L_{i+1}-L_{i}) \end{array}$$

As we can see from Equation (9), the more densely sampled our reference points are, the more precise our interpolated image position will be. In our experimental setup, because the navigation platform is moved slowly toward the wall, for any pair of nearby reference points *P_i_*(*L_i_,x_i_,y_i_*),*P_i_*_+1_(*L_i_*_+1_*,x_i_*_+1_*,y_i_*_+1_) the constraints (10) are maintained, which keeps the interpolation error within one pixel:

(10) $$\{\begin{array}{c} |x_{i}-x_{i+1}|<1\\ |y_{i}-y_{i+1}|<1 \end{array}$$

#### 3.2.2. Geometrical Calibration of LDM and Camera

**Figure 9.**
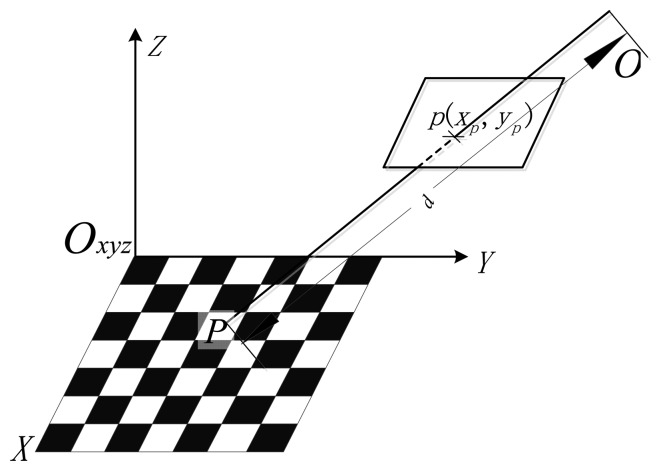
The combined calibration setup.

A flat panel with grid corners is all that is necessary for the geometrical calibration. We take images from different positions on the panel with synchronized measurements from the LDM. For brevity, in the following account, each exposure and its associated measurement is referred to as a “shot” [18]. The calibration sequence and the calculation of geometrical parameters *B* and *θ* are summarized as follows:

A reference system is defined in the panel such that regular grid corners are assigned coordinates (see Figure 9). We assume the panel to be completely flat; all the reference points have the same *Z* coordinate of 0.

(1) For every shot, we need to know the camera coordinates *T*(*X_o_,Y_o_,Z_o_*) and its rotations *R*(*φ_o_*,*ω_o_*,*κ_o_*) with reference to the panel. As the camera parameters have been calibrated, we use the same calibration package [22] to detect the grid corners and then compute extrinsic parameters only. An estimate of uncertainty of these parameters can also be obtained.
  From the index table created above, the projection of the laser pointer *p*(*x_p_*,*y_p_*) is also known from its synchronized image calculated using Equation (11). In Figure 9, the vector $\vec{Op}$ can be described by the following equation:
  (11) $$\{\begin{array}{c} R=\left[\begin{array}{ccc} 1 & 0 & 0\\ 0 & \cos\varphi & -\sin\varphi \\ 0 & \sin\varphi & \cos\varphi \end{array}\right]\;\left[\begin{array}{ccc} \cos\omega & 0 & \sin\omega \\ 0 & 1 & 0\\ -\sin\omega & 0 & \cos\omega \end{array}\right]\;\left[\begin{array}{ccc} \cos\kappa & -\sin\kappa & 0\\ \sin\kappa & \cos\kappa & 0\\ 0 & 0 & 1 \end{array}\right]\\ \vec{Op}=R•\kappa^{-1}•\left[\begin{array}{c} x_{p}\\ y_{p}\\ 1 \end{array}\right]+T \end{array}$$
  Here, *K*^−1^ is the inverse of the camera intrinsic matrix. By intersecting $\vec{Op}$ with the panel, we obtain the laser pointer *P*(*X_p_,Y_p_,Z_p_*).The distance *d* can then be calculated:
  (12) $$d=\sqrt{{\left(X_{P}-X_{O}\right)}^{2}+{\left(Y_{P}-Y_{O}\right)}^{2}+{\left(Z_{P}-Z_{O}\right)}^{2}}$$
(2) From Equation (1), it is clear that we require only two shots to compute geometrical parameters theoretically. For example, for any two shots with laser measurements *L*_1_ and *L*_2_, we can calculate the corresponding laser pointer distances *d*_1_, *d*_2_ to the camera center following the steps above. The geometrical parameters can then be calculated:
  (13) $$\{\begin{array}{c} B=\sqrt{\left(L_{1}•({L_{2}}^{2}-{d_{2}}^{2})-L_{2}•({L_{1}}^{2}-{d_{1}}^{2}))/(L_{2}-L_{1}\right)}\\ \theta =\cos^{-1}((B^{2}+{L_{1}}^{2}-{d_{1}}^{2})/(2•L_{1}•B)) \end{array}$$
  However, instead of taking all the shots into consideration, we choose shots providing good data by running a RANSAC scheme. Here, the error function *f*(*L*,*d*) for every laser pointer *j* is defined to be:
  (14) $$f(L_{j},d_{j})=\left|\sqrt{{L_{j}}^{2}+B^{2}-2•L_{j}•B•\cos\theta }-d_{j}\right|,j=1,2,\ldots ,N$$
  In our experiment, the error threshold is set to be 1 cm and error values below this threshold are grouped as inliers. Using this threshold setting, *B* and *θ* can be recovered robustly, rejecting those shots with great uncertainty.
(3) Step 3 provides us with initial values for the extrinsic parameters and the camera shots that provide good data. These parameters are further refined by minimizing the error calculated from Equation (14), using the Levenberg-Marquardt method. Generally, convergence is reached within three to four iterations and the squared mean error is 2 mm, nearly the same accuracy as that of the LDM.

## 4. A Global Scaled Monocular VO Algorithm for Astronaut Navigation

Once all the calibration steps mentioned above are completed, we are ready for astronaut navigation. This navigation framework, as illustrated in Figure 4, can be seen as an extension to traditional monocular VO. We will present the algorithm in three parts. The first part deals with robust motion estimation, while the second part deals with laser pointer tracking on image. The final part deals with the scale ambiguity problem, including relative scale calculation and global scale correction with the aid of laser measurements.

### 4.1. Robust Relative Motion Estimation

**Figure 10.**
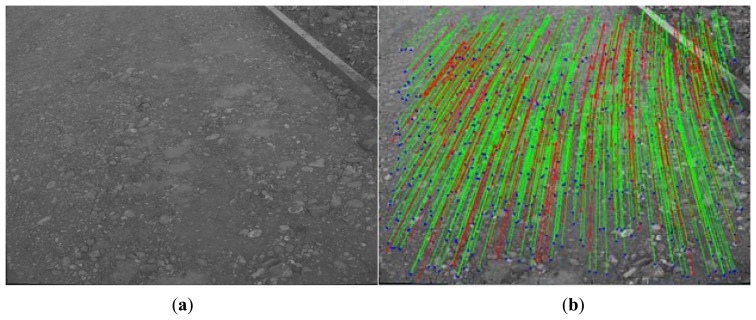
(**a**) Original key-frame selected from the dataset; (**b**) Feature tracking between nearby key-frames (previous dotted in green, current dotted in blue), stereo matches (green), and triple matches (red).

The core of VO is robust estimation of frame-to-frame motion. This is a classical problem, and a large number of solutions have been proposed to solve this issue [26]. Most of these works use RANSAC for outlier rejection. In our work, we choose to integrate a variant of RANSAC, named PROSAC [27], to remove outliers more effectively. As is reported by Chum [27], PROSAC exploits the linear ordering defined on the correspondences by a similarity function instead of treating all correspondences equally. In our experiments, it can detect outliers more effectively than RANSAC, while achieving large computational savings. The relative motion is then computed by 2D-to-2D motion estimation. Iterative refinement is also performed to obtain more accurate results by using all the inliers. We summarize the steps in the robust motion estimation algorithm as follows:

(1) Image features are detected using the Shi-Tomasi corner detector [28,29]. These features are further refined to reach sub-pixel localization accuracy [29,30]. As the images are taken from nearby viewpoints, instead of detecting features individually in images and then matching them, we find features in one image and track these features in the succeeding images by searching in the nearby neighborhood. We take an implementation called the Kanade-Lucas-Tomasi (KLT) tracker [31] to track features over long image sequences. Mutual consistency checking is also undertaken to remove false matches. As the distribution of features has been reported to affect VO results [5,8], the image is portioned into 10 buckets by 10 buckets and the detector is applied to each cell with a threshold of a maximum number of features set in each bucket.
(2) Key-frames are selected automatically based on the number of features tracked. A criterion is set up such that a new key-frame *I_i_* is introduced whenever the number of stereo matches with the last key-frame *I_i_*_+1_ is below *M*_1_ [11]. Additionally, a key-frame is introduced whenever the number of triple matches with key-frame *I_i_*_−2_ is below *M*_2_ (in our experiment, we set *M*_1_ = 1000 and *M*_2_ = 300), as shown in Figure 10. After this key-frame is selected, mutual consistency checking is performed again between *I_i_* and *I_i_*_+1_ in case of tracking drift issues [32].
(3) The essential matrix between frame *I_i_* and *I_i_*_−1_ is estimated using the 5-point algorithm [33] and PROSAC followed by matrix factorization into rotation *R* and unitary translation *T* using Horn's method [34].
(4) The refinement of *R* and *T* is further refined by minimizing the reprojection error using the Levenberg-Marquardt nonlinear optimization.

Currently, our motion estimation is simple for the selection of key-frames, by making the assumption that the distribution of features is in a good state in the image and the number of features tracked from the last key-frame falls off gradually when subsequent frames arrive. Therefore, a threshold can be set to restrict the track length as in step 2. In particular, the number of triple matches involving the last two key-frames is constrained for further calculation of relative scale between nearby image pairs. However, this assumption fails when rapid cornering occurs, which is not taken into consideration in our current VO scheme and was avoided in the experiment.

### 4.2. Laser Pointer Matching

**Figure 11.**
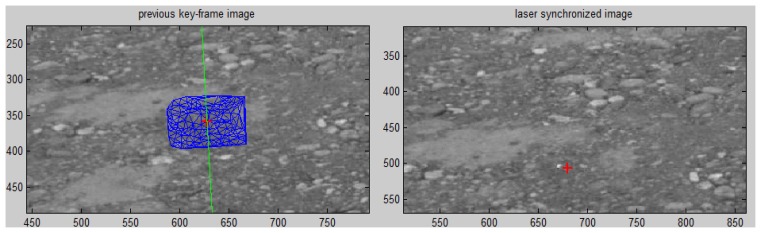
The laser pointer projection (right image, marked in red) is matched on the key-frame (marked in red) through disparity consistency constraints by Delaunay triangulation (blue), checked by the laser pointer's conjugate epipolar line (green).

Suppose that at time *t_n_* during the navigation stage, we obtain the laser pointer *P*'s distance measurement *L* and synchronized image frame *C_n_*. We can easily obtain the projection of the laser pointer *p* on *C_n_* from the index table created during calibration. As previously mentioned, we need to match *p* in the previous key-frame and the next key-frame to correct scale drift. The difficulty lies in that the projection often does not belong to feature points that can be detected by feature detectors, and it is difficult to match *p* in other frames with commonly used matching techniques. In our work, a coarse-to-fine matching method is proposed to deal with this difficulty. Epipolar constraints are also taken into consideration for robustness. The principle of this coarse-to-fine matching method is as follows:

(1) A window centered on *p* with a radius of 50 pixels is used to select feature points located in this window from frame *C_n_*. Here, we denote this sub-image as *I*_1_. As the correspondences of these feature points with the nearby key-frame are known during feature tracking, we can compute the disparity of these feature point with reference to this key-frame. A Delaunay triangulation is constructed for this set of selected feature points. By searching in the Delaunay triangulation, a triangle containing *p* can be found. For the three vertex points of the triangle, we establish the affine transformation from the coordinates of these three points in frame *C_n_* to the nearby key-frame. By solving the affine transformation parameters, we can obtain *p*'s transformed coordinate, *p*_1_ in the nearby key-frame.
(2) A window centered on *p*_1_ with a radius of 50 pixels is constructed in the nearby key-frame. We denote this sub-image as *I*_2_. More feature points are detected by lowering the response threshold of the feature in *I*_1_ and *I*_2_. Thus, dense feature points can be matched, followed by RANSAC to remove outliers. A similar step is performed by constructing a new Delaunay triangulation to calculate *p*'s transformed coordinate, *p*_2_. By using constraints with dense feature points, *p*_2_ becomes closer to the laser pointer projection in the nearby key-frame.
(3) These steps help us to find a good initial position. Then we can use dense tracking techniques to calculate the final position with further refinement using Horn's method [35]. In our experiment, we set the search range to be 3 pixels, which greatly decreases false matches by constraining the search range to within a small area.
(4) Now we obtain the refined image position on the nearby key-frame after performing the last three steps successfully. To increase the robustness of this coarse-to-fine method, the final refined position on the key-frame is verified using epipolar constraints performed as below:

For the laser pointer projection in frame *C_n_*, we can calculate its corresponding epipolar line on the nearby key-frame. This final position is accepted as valid when the distance to this epipolar line is within one pixel, as shown in Figure 11.

The principle of the coarse-to-fine matching scheme is robust when choosing a good initial position as close as possible to the laser pointer's projected position on the nearby key-frame, thus decreasing the possibility of choosing false matches that also have similar image patches. Then subsequent local dense matching techniques can be performed effectively within a small area.

### 4.3. Robust Scale Estimation

Although we obtain the transformation relationship *T_i_*_,_*_i_*_+1_ between the image pair {*i*,*i*+1} through motion estimation, we need to concatenate *T_i_*_,_*_i_*_+1_ with the previous transformation *T_i_*_−_*_1_*_,_*_i_* estimated from image pair {*i*−*1*,*i*} to recover the trajectory of the whole image sequence. When laser pointer projection is successfully matched, we can scale this relative model using the global distance. Otherwise, we use the relative scale calculation with the triple match constraint.

#### 4.3.1. Computation of Relative Scale

For monocular image sequences, a proper relative scale must be calculated when the absolute scale of the transformation cannot be computed without extra absolute scale information. Triple matches across three key-frames are required to calculate this relative scale [36]. One simple way of doing this [37] is to triangulate two 3-D points, *X_m_* and *X_n_*, from image pairs {*i*,*i*+1} and {*i*−*1*,*i*}, then the relative scale can be determined from the distance ratio between point pairs in subsequent image pairs as follows:

(15) $$r=\frac{\left\|X_{m,\{i,i+1\}}-X_{n,\{i,i+1\}}\right\|}{\left\|X_{m,\{i-1,i\}}-X_{n,\{i-1,i\}}\right\|}$$

We can see from Equation (15) that at least two features need to be matched across three frames. For robustness, scale ratios for many point pairs are computed and the median values are often chosen in case of outliers. In our experiment, nearly 300 features across three frames are kept for this relative scale calculation, which is thus robust against gross errors.

#### 4.3.2. Correction of Scale Drift with Global Scale Constraints

As monocular VO works by computing the camera route incrementally, the path errors are bound to grow over time with the accumulation of frame-to-frame motion. In our navigation task, it is important to keep path drift as small as possible. Though bundle-adjustment-based monocular VO has been proved to decrease the path drift effectively, it still suffers from scale drift. In our work, we concentrated on the correction of scale drift through combination with a laser distance meter. We have given a brief note on the principle of our method in Figure 3. The detailed steps in the correction of the scale drift are summarized as follows:

(1) When a new key-frame is introduced, laser pointer tracking is performed on this new key-frame and its previous key-frame.
(2) If laser pointer matching fails, we return to the traditional relative scale calculation. Otherwise, supposing that this laser pointer is collected at time *t_n_* with laser meter measurement *L_n_*, synchronized frame *C_n_* and nearby key-frame pairs *C*_1_ and *C*_2_, we obtain the projection of the laser pointer *p_n_* on *C_n_* using the index table and the projections *p*_1_ on *C*_1_ and *p*_2_ on frame *C*_2_ through laser pointer matching. As we obtain the transformation relationship *T* using the frame pairs *C*_1_ and *C*_2_, we can obtain the 3-D position of the laser pointer *P_n_* by triangulating *p*_1_ and *p*_2_. When multiple laser pointers are matched successfully, we select the one with the maximum intersection angle. Most of the time, *C_n_* is between key-frame pairs, as illustrated in Figure 3. However, when it is exactly the new key-frame, we only need to match the laser pointer on the previous key-frame.
(3) By triangulating image pairs between *C*_1_ and *C*_2_, we can obtain a series of 3-D points. As we also know the projection of the images of these 3-D points on *C_n_*, we can obtain the position of the camera *P_c_* at time *t_n_* by solving the PnP (Pose from n Points) problem [37]. Meanwhile, the global distance *d_n_* from the laser pointer to the centre of the camera at time *t_n_* can be calculated from Equation (1) with *L_n_* and calibrated geometrical parameters. Thus, we can calculate the global scale as follows:
  (16) $$r=\frac{d_{n}}{\left\|P_{n}-P_{c}\right\|}$$
  We can see from the above that scale drift can be corrected whenever the laser pointer is successfully matched on the key-frame pair. When laser pointer matching fails, we can use relative scale calculation, introducing scale drift when these relative scales accumulate until the next laser point is matched again. In this way, our enhanced monocular VO corrects the scale drift over a certain number of frames, effectively reducing the final position drift.

## 5. Experiments and Results

In this section, field tests using the proposed enhanced monocular VO algorithm are carried out in simulated lunar environments. These tests are designed to validate the feasibility and effectiveness of the proposed enhanced monocular VO method. The camera we used has a field of view of 30° and is rigidly attached to a LDM while facing forward during walking. The laser frequency was set at 1 Hz and the camera was set at 10 Hz, meaning that we have the distance measurements with synchronized captured images every second. The person carrying the system walked at a velocity of about 1.0 m/s. Here we report two typical experiments on outdoor and simulated lunar environments.

**Figure 12.**
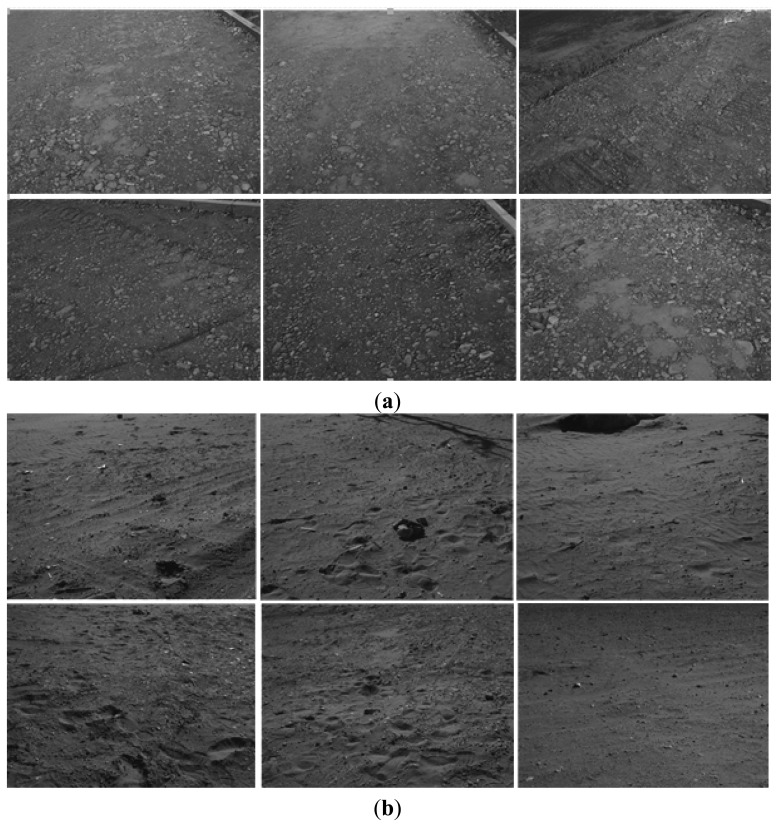
(**a**) Sample images from the first dataset at a construction site; (**b**) Sample images from the second dataset in a desert.

For the first dataset (see Figure 12a), 1108 frames and 89 laser pointer measurements (when the distance signal does not return in a valid time, this measurement is dropped in our system) were taken at a construction site covering a total distance of approximately 110 m. Finally, 173 key-frames were selected automatically and 41 key-frame pairs were successfully matched with the laser pointer projection. We can see from Figure 12a that the soil hardness is not as soft as that of the lunar surface and rocks of various sizes are spread across the terrain surface, which make features quite easy to detect among the two datasets. Therefore the first dataset can be taken as an ideal dataset to evaluate the performance of proposed VO method compared to traditional one. The second dataset was taken in a desert which seems to be more similar to the lunar surface. For the second dataset, there are 3840 frames and 368 laser pointer measurements covering a total distance of 300 m, of which 402 key-frames were selected and 154 key-frame pairs found laser pointer's projection. We can see from Figure 12b that the sandy surface in this dataset is more similar to the type of the lunar surface than the former dataset, with footprints clearly seen on this soft sandy terrain. Therefore, we can take it as a large-scale outdoor test field to simulate the real lunar terrain.

We walked a loop with the origin set at [0,0]. The same image was used for the first and last positions to ensure that the true last camera pose was exactly the same as where the first image was recorded. The commonly used approach called sliding window bundle adjustment is not involved in our current monocular VO scheme, nor is the loop closure correction.

**Figure 13.**
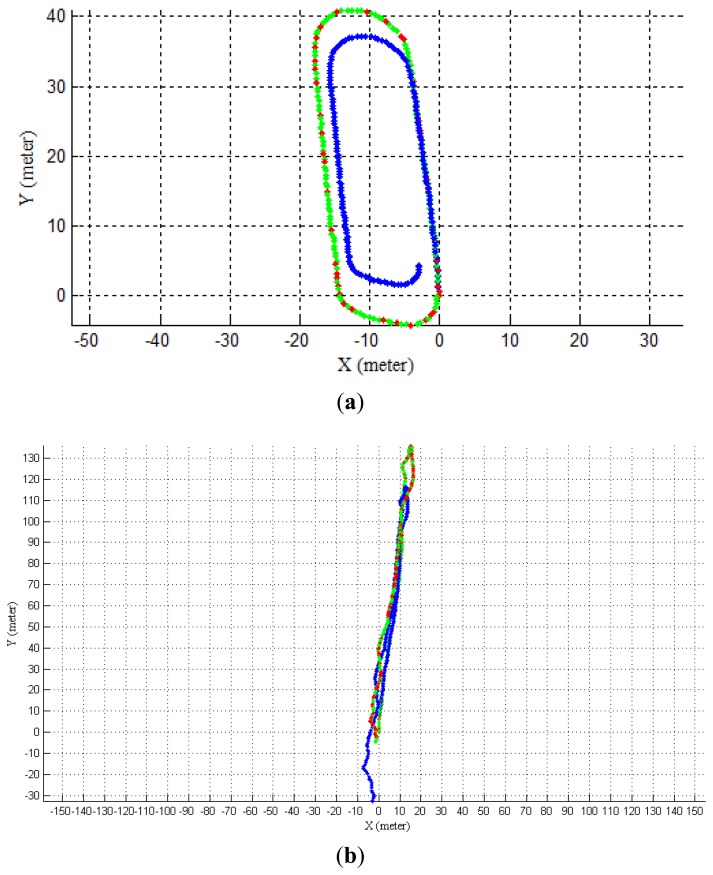
Estimated trajectory using monocular VO (blue); estimated trajectory using enhanced monocular VO (green); red dots represent frames when the synchronized laser pointer was successfully matched and scale correction was taken. (**a**) the first dataset; (**b**) the second dataset.

Given that the loop is closed, we can use it to measure the navigation accuracy. Figure 13 shows the final result of our enhanced monocular VO scheme compared with the traditional scheme. The motion estimates of the first key-frame pair are both globally scaled with the LDM to facilitate comparison. From Figure 13a, obvious improvement can be seen with the relative error decreased from 5.91% to 0.54% for the first dataset when the LDM is added. In Figure 13b, the relative error decreased from 12.02% to 1.71% for the 300 m route in the desert. Considering the second dataset's high similarity to the lunar surface, it can be inferred that this enhanced monocular VO scheme should also work well when dealing with real lunar environment. Moreover, it can be inferred from Figure 13b the longer we walked, the higher the improvement is as scale drift accumulates severely for a single camera. As we emphasize the scale drift issue, the relative transformation relationships of key-frames are kept the same for the enhanced VO and the traditional VO except for the difference in scale selection during the whole trajectory, which shows that the accuracy is improved significantly by a better scale selection scheme.

**Figure 14.**
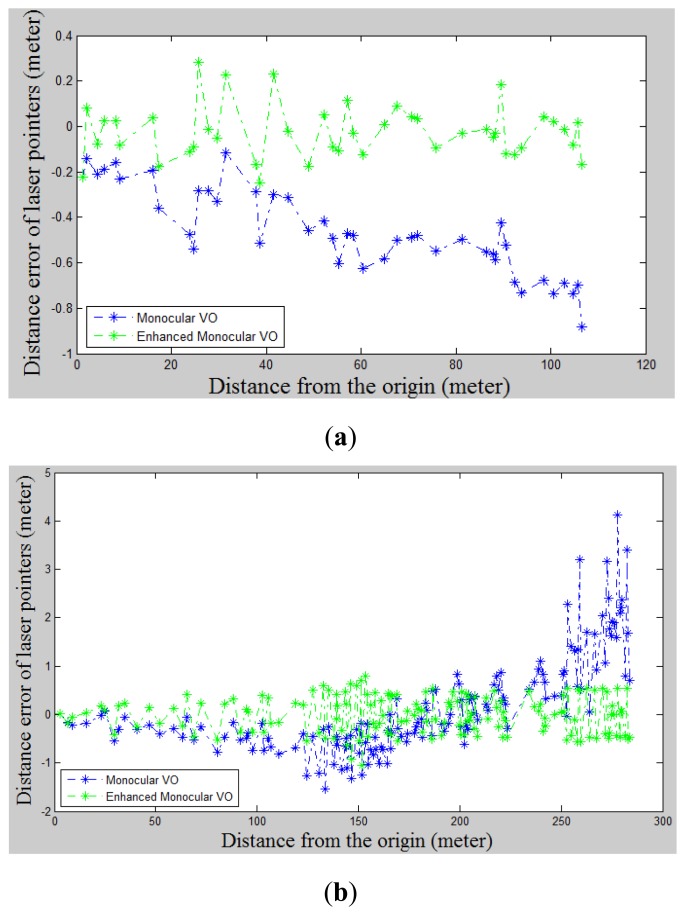
The distance error of the laser pointer with our enhanced monocular VO scheme compared to the traditional one. (**a**) the first dataset; (**b**) the second dataset.

We also compared the distance errors of laser points between LDM-aided monocular VO and the traditional one with the distance travelled. By taking the distance calculated from Equation (1) as a reference, we triangulate the laser pointer's projections in key-frame pairs in both VO schemes, as illustrated in Figure 14. As the distance constraints of the first laser pointer are used in both VO schemes, this error is set as zero in the beginning. It is clear from Figure 14 that the gradually accumulated scale drift is corrected effectively with our VO scheme.

## 6. Summary and Conclusions

In this paper, we have presented an enhanced monocular VO scheme to resolve the scale drift with the aid of LDM. We concentrated on the integration of LDM with monocular camera mounted on a walking person modeling astronaut navigation on a simulated lunar surface. A robust and simple extrinsic calibration method has been proposed. Based on this method, for every laser point measured, its projected image position and the distance to synchronized camera center is also precisely known. Later, an enhanced monocular VO scheme was proposed by integrating measurements from LDM. Accurate results for approximately 110 m of walking at a construction site were demonstrated by correcting the scale drift, outperforming the traditional monocular scheme by almost a factor of ten. Further experiments were taken in a desert to validate our method's feasibility and robustness on simulated lunar terrain compared to traditional one.

One of the most remarkable differences between our monocular VO scheme and previous methods is the introduction of LDM to correct the scale drift. Scale error propagation over time is avoided effectively, demonstrating the strength of LDM in the field of monocular VO. In our current system, the commonly used BA technique is not used. In the future, the sliding window BA with measurements using LDM will be integrated into our system, further improving the pose drift and being more practicable for astronaut long term navigation.
